# The role of eIF5A in epidermal growth factor-induced proliferation of corneal epithelial cell association with PI3-k/Akt activation

**Published:** 2011-01-06

**Authors:** Lan Ding, Ling-Juan Gao, Ping-Qing Gu, Shu-Yu Guo, You-Qun Cai, Xing-Tao Zhou

**Affiliations:** 1 Department of Ophthalmology, EENT Hospital, Fudan University, Shanghai, P.R. of China; 2 Clinical Laboratory, Nanjing Maternity and Child Health Care Hospital, Nanjing, P.R. of China; 3 John Curtin School of Medical Research, The Australian National University, Canberra ACT, Australia

## Abstract

**Purpose:**

After excimer laser surgery, epidermal growth factor (EGF) plays an important role in injured corneal epithelial cell on myofibroblastic cell formation in corneal stroma. The purpose of the study is to investigate the precise mechanism of EGF on corneal wound healing, particularly on epithelial proliferation and migration.

**Methods:**

In this study we applied small interference RNA (siRNA) to knock down the expression of eukaryotic translation initiation factor 5A (eIF5A) in corneal epithelial cells. The relative mRNA and protein expression of matrix metallopeptidase 9 (MMP9) and proliferating cell nuclear antigen (PCNA) was determined via real-time PCR and western blot analysis. The proliferative potential of EGF was evaluated via a proliferation assay using the measurement of ^3^H-thymidine incorporation (^3^H-TdR). HCEpiC apoptosis was subjected to flow cytometric analysis.

**Results:**

The results showed eIF5A expression was enhanced and there was a statistically significant increase in EGF treatment compared to the control group. Real-time PCR, western blot analysis, and the proliferation assay demonstrated significantly lower MMP9 and PCNA expression and proliferation cell counts in *eIF5A* siRNA-treated groups versus significantly higher levels in *EGF* plus *eIF5A* siRNA-treated groups. The data analysis showed that *eIF5A*, *MMP9*, and *PCNA* expression decreased as a result of the inhibitor LY294002. Apoptotic cells were increased in the *EGF* plus *eIF5A* siRNA, *EGF* plus LY294002, and *EGF* plus *eIF5A* siRNA plus LY294002 groups as compared with the EGF siRNA group.

**Conclusions:**

These results indicate that EGF-induced upregulation of *eIF5A* stimulates corneal epithelial cell proliferation in vitro. EGF stimulation of corneal epithelial proliferation was through the phosphatidylinositol 3-kinase (PI3-k)/protein kinase B (Akt) signaling pathway.

## Introduction

Photorefractive keratectomy (PRK) can result in tissue ablation with a high degree of precision and minimal damage to the adjacent tissues, and this has become a favored operation to correct refractive errors, including myopia, hyperopia, and astigmatism [1]. The operation is effective and safe, but some patients develop a postoperative wound-healing response, causing visual impairment.

The first stage of wound healing in the cornea after PRK is epithelial migration. The maintenance of the normal corneal epithelial thickness and its protective function depends on a balance between the basal layer cell proliferation at a rate adequate to replace terminally differentiated cells in the superficial layers [2]. In a variety of studies, growth factors have been identified to be involved in maintaining epithelial renewal or epithelial cell proliferation. In particular, epidermal growth factor (EGF) is a substantive contribution involved in this renewal process through its role in stimulating proliferation and differentiation [3]. An optimal dose of EGF present in the medium can repair the wound of the corneal epithelial cells at an enhanced rate. EGF stimulates wound healing in vitro by a myriad of methods. One of the events in these cascades includes the inactivation of apoptotic factors, which in turn triggers proliferation in corneal epithelial cells [4].

Eukaryotic translation initiation factor 5A (eIF5A) is highly conserved in eukaryotes and is the only known protein containing the unusual spermidine-derived amino acid residue hypusine [5]. Early observations of a correlation between hypusine synthesis and cell growth suggested an important role for hypusine in cell proliferation [5,6]. eIF5A may be a bimodular protein interacting with both RNA and proteins and is presumed to have an important role in the translation machinery [7]. Although eIF5A is intimately involved in eukaryotic cell proliferation, the true physiologic function of this essential factor has yet to be elucidated, and the potential role of eIF5A needs further investigation.

In this report we present important results to demonstrate that eIF5A upregulation is associated with EGF induction of proliferation via phosphatidylinositol 3-kinase (PI3-k)/protein kinase B (Akt) activation. Apparently, EGF-induced human corneal epithelial cell (HCEpiC) proliferation requires upregulation of eIF5A to promote premature differentiation.

## Methods

### Reagents used in experiments

The human corneal epithelial cell (HCEpiC) line was obtained from the Center of China Type Culture Collection (Wuhan, China). Lipofectamine 2000 was purchased from Invitrogen (Carlsbad, CA). Small interference RNA (siRNA) was synthesized by Wuhan Genesil Biotechnology Co., Ltd (Wuhan, China). Human epidermal growth factor (EGF; 10 ng/ml) and LY294002 (PI3-k inhibitor) were obtained from Sigma-Aldrich Inc. (St. Louis, MO). Phototope-horseradish peroxidase (HRP) western blot detection system, including antimouse immunoglobulin, HRP-linked antibody, biotinylated protein ladder, 20× LumiGLO reagent, and 20× peroxide were purchased from Cell Signaling Technology (Beverly, MA). The annexin V- fluorescein isothiocyanate (FITC)/propidium iodide flow cytometry assay kit was purchased from Invitrogen. The antibodies for anti- eukaryotic translation initiation factor 5A (eIF5A), matrix metallopeptidase 9 (MMP9), proliferating cell nuclear antigen (PCNA), anti-phospho-Akt, and anti-total Akt antibodies were products of Cell Signaling Technology. Cell culture supplies were purchased from GIBCO/Life Technologies Inc. (Gaithersburg, MD). The ^3^H-thymidine was endowed by the isotope laboratory of Nanjing Medical University. Unless otherwise specified, all other reagents were of analytical grade.

### Human corneal epithelial cell line culture and small interference RNA transfection

HCEpiCs were cultured in Dulbecco’s Modified Eagle’s Medium/Ham’s F-12 medium (Sigma-Aldrich Inc.) containing 10% fetal bovine serum and 5 μg/ml insulin in a 37 °C incubator gassed with 5% CO_2_. Cells used in experiments were from five to seven passages.

Lipofectin transfection of *eIF5A* siRNA was performed according to the vendor’s protocol: 500 pmol of eIF5A siRNA and 10 μl lipofectin were diluted in 750 μl of OptiMEM (Gibco BRL Life Technologies) in one well. After pre-incubation for 45 min at 37 °C, both solutions were mixed and incubated for an additional 15 min at room temperature. The lipofectin/*eIF5A* siRNA mixture was subsequently overlaid onto the HCEpiCs and incubated for 2 h. Finally, 1 ml of growth medium (20% fetal calf serum) per well was added for further cultivation of the HCEpiCs.

### Construction of *eIF5A* small interference RNA-expressing plasmid vector

In this experiment, the targeted siRNA sequences for *eIF5A* were 5′-AAC GGA ATG ACT TCC AGC TGA-3′. Using pGenesil-1 as the vector backbone, the vectors of *eIF5A* siRNA-expressing plasmid were constructed by using green fluorescent protein (GFP) as the reporter gene. Near the 5' end of the two oligonucleotides, a BamHI and HindIII restriction site overhangs; a 6 nucleotide poly (T) tract recognized as a RNA pol III termination signal is located at the 3' end of the siRNA template. The synthesized and annealed siRNA was ligated into the BamH1 and Hind 3 site of the pGenesil-1 expression vector. An unrelated gene siRNA was chosen as a negative control.

### Western blot analysis

The HCEpiCs were collected with sample buffer after specific treatments. HCEpiCs were incubated in lysis buffer containing 20 mM Tris-HCl (pH 7.5), 1% Triton X-100, 1 mM ethylene diamine tetracetic acid (EDTA), 150 mM NaCl, 10% glycerol, 1 mM Na_3_VO_4_, 50 mM NaF,1 mM phenylmethyl sulfonylfluoride (PMSF), and protease inhibitor for 20 min on ice. After insoluble debris was precipitated by centrifugation at 13,000× g at 4 °C for 15 min, the supernatants were collected and assayed for protein concentration using the Bradford method. An equal amount of protein per sample (15 µg) was resolved on 10% sodium dodecyl sulfate polyacrylamide gel electrophoresis and transferred onto a polyvinylidene fluoride (PVDF) membrane. The transferred membranes were blocked for 1 h in 5% nonfat milk in PBS containing 135 mM NaCl, 2.7 mM KCl, 1.5 mM KH_2_PO_4_, and 8 mM K_2_HPO_4_, pH 7.2, and 0.05% Tween-20 and incubated with appropriate primary antibodies and HRP-conjugated secondary antibodies. The protein bands were visualized using the enhanced chemiluminescence western detection system.

### Cell proliferation assay

HCEpiCs were plated into 96-well plates and incubated overnight. The cultures were then serum starved for 24 h and treated with experimental agents for another 24 h. DNA synthesis was determined by ^3^H-thymidine incorporation(^3^H-TdR) for the final 18 h. The media were carefully removed and the cells detached with 50 μl trypsin-EDTA. The cells were then harvested onto glass filters with a Tomtech cell harvester (LKB Wallac, San Francisco, CA), and the radioactivity retained on the dried filters was measured by the addition of 50 ml scintillation liquid and counted in a TopCount NxT scintillation counter (LKB Wallac).

### Detection of apoptotic cells

HCEpiCs apoptosis were measured on a Coulter Epics XL flow cytometer (FCM, Beckman Coulter, Erembodegen, Belgium) with apoptosis cells being annexin V positive/propidium iodide (PI) negative. HCEpiCs in a 6-well plate with a density of 1×10^6^ were harvested and washed once with ice-cold PBS. HCEpiCs were resuspended with binding buffer (10 mM HEPES [pH 7.4], 140 mM NaCl, 2.5 mM CaCl_2_) before being transferred to a 5-ml tube. Then cells were stained with 5 ml of annexin V-FITC for 30 min and 5 ml PI (10 μg/ml) for 15 min in the dark at room temperature. Cellular DNA was detected by FCM and apoptosis rate was computed. Annexin V and PI staining were yielded equivalent results. Data from duplicates were averaged and used as a single representation of the percentage of apoptotic cells for any given treatment.

### Statistical analysis

Results are presented are presented as means±standard deviation. Differences between various data sets were tested for significance using the Student *t* test, and p values less than 0.05 were considered significant (*p<0.05; **p<0.01).

## Results

### Expression of *eIF5A* induced by EGF in HCEpiCs

**Figure 1:**
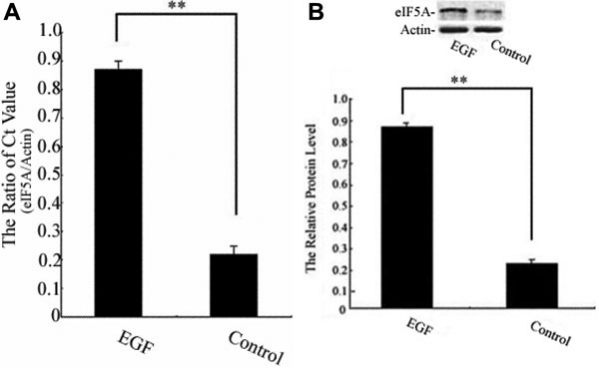
Eukaryotic translation initiation factor 5A (*eIF5A*) expression in human corneal epithelial cells (HCEpiC) was assessed in this experiment. **A**: Relative *eIF5A* gene expression levels at 18 h after administration of epidermal growth factor (EGF; 10 ng/ml) and medium alone (control) was detected by real-time polymerase chain reaction (PCR). The amplification plot of *eIF5A* and β-actin (*ACTB*) are shown. The graph shows the relative eIF5A protein levels normalized to β-actin. (**) were highly significant when compared with control group, in both cases p-value <0.01. **B**: The expression of eIF5A protein was measured by using western blot analysis. The graph shows the relative eIF5A protein levels normalized to β-actin. Results are presented as the mean±standard deviation of three independent experiments (n=3), each conducted in triplicate. (**) were highly significant when compared with control group, in both cases p-value <0.01.

To investigate the relationship between the effects of EGF on the production of eIF5A in HCEpiCs, the expression of *eIF5A* in HCEpiCs treated with EGF (10 ng/ml) was assessed (Figure 1A,B). The results showed that the expression of *eIF5A* was significantly increased (p<0.01) in HCEpiCs treated with EGF when compared with the control (medium alone). This suggests that *eIF5A* may play an important role in the growth of EGF-treated HCEpiCs.

### Role of *eIF5A* on EGF-induced expression of *MMP9*, *PCNA*, and HCEpiC proliferation

**Figure 2:**
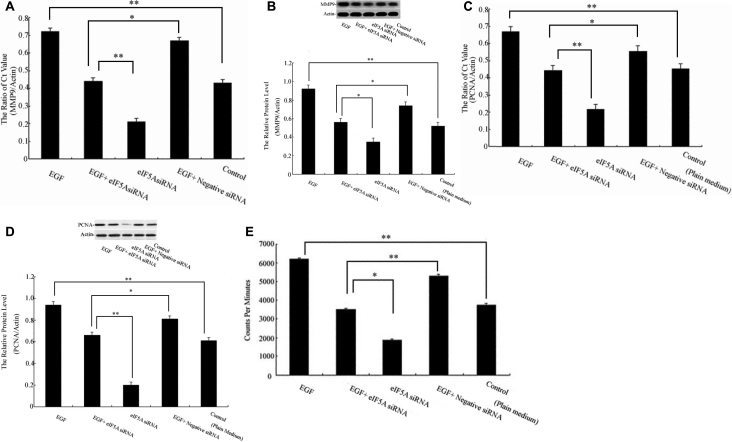
Effects of *eIF5A* on matrix metallopeptidase 9 (*MMP9*), proliferating cell nuclear antigen (*PCNA*), and the proliferation of HCEpiCs were assessed in this study. Cultured HCEpiCs were treated with EGF, EGF+*eIF5A* siRNA, *eIF5A* siRNA, EGF+negative siRNA, and medium alone (control) for the indicated times. Data were expressed by mean±SD of three independent experiments.** A**-**B**: Relative expressions of *MMP9* mRNA and protein level at 24 h after administration of different treatments were detected by real-time PCR and western blot, respectively. The graph shows the relative eIF5A protein levels normalized to β-actin; data are expressed as mean±standard deviation (SD) of three independent experiments. (**) were highly significant when compared with control group, in both cases p-value <0.01, asterisks indicate p-value <0.05 with the Student *t* test. **C**-**D**: Relative expressions of *PCNA* mRNA and protein level at 36 h after administration of different treatments were detected by real-time PCR and western blot, respectively. The graph shows the relative eIF5A protein levels normalized to β-actin. (**) were highly significant when compared with control group, in both cases p-value <0.01, asterisks indicate p-value <0.05 with the Student *t* test. **E**: HCEpiC proliferation at 48 h after administration of different treatments was detected by ^3^H-TdR. Data were expressed as mean±SD of three independent experiments. **p<0.01 versus control group, asterisks indicate p<0.05 in *t* test.

The role of eIF5A on the survival of EGF-treated HCEpiCs was investigated. Using real-time PCR and western blot analysis, the expression for *MMP9* mRNA and protein was demonstrated in Figure 2A,B. Statistically significant (p<0.01) increases compared to control group were noted in the EGF group; statistically significant (p<0.01, p<0.05 respectively) changes compared to EGF + eIF5A siRNA were noted in eIF5A siRNA and EGF + negative siRNA. Additionally, our analysis showed that the expression cell cycle-associated protein *PCNA* mRNA and protein expression in EGF, EGF + eIF5A siRNA, eIF5A siRNA, EGF + negative siRNA, and medium alone group was measured in Figure 2C,D. Statistically significant (p<0.01, p<0.05 respectively) differences in mRNA and protein expression as compared to the EGF + eIF5A siRNA group were noted in the eIF5A siRNA and EGF + negative siRNA groups. The results of densitometric analyses demonstrated that HCEpiCs exposed to EGF exhibited increased *MMP9* and *PCNA* expression when compared to the control group.

The proliferation of HCEpiCs was assessed by the changes in DNA synthesis. Figure 2E shows that EGF could significantly upregulate DNA synthesis as compared with medium alone. There was a 33% decrease in DNA synthesis in HCEpiCs exposed to treatment with EGF+*eIF5A* siRNA compared to EGF+negative siRNA and an approximately 45% decrease with *eIF5A* siRNA treatment compared to EGF+*eIF5A* siRNA for 48 h after the initial treatment. This finding suggests that *eIF5A* may play an important role in the survival of EGF-treated HCEpiCs.

### Effect of *eIF5A* on EGF-induced PI3-k/Akt activation in HCEpiCs

**Figure 3:**
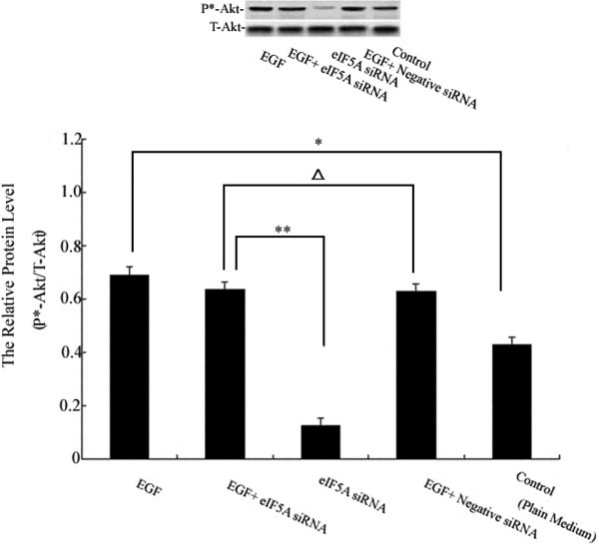
The effect of *eIF5A* treatment on phosphorylation of protein kinase B (Akt) in HCEpiC was detected in this experiment. Western blot analyses of phospho-Akt in EGF, EGF+*eIF5A* small interference RNA (siRNA), *eIF5A* siRNA, EGF+negative siRNA, and medium alone (control)-treated cells. At 48 h post infection, HCEpiCs were stimulated with EGF. After further incubation for 30 min, cells were subjected to western blot analysis. The graph shows the relative phospho-Akt protein levels normalized to total-Akt. (**) were highly significant when compared with control group, in both cases p-value <0.01, asterisks indicate p-value < 0.05 with the Student t test.

The effect of *eIF5A* on EGF-treated HCEpiCs may be involved in various signaling pathways, but which signaling pathway plays a main role is unclear. In this experiment we focused on whether the activation of the PI3-k/Akt signaling pathway in HCEpiCs needed eIF5A stimulation. Figure 3 shows that a change in phospho-Akt occurred after EGF, EGF+*eIF5A* siRNA, *eIF5A* siRNA, EGF+negative siRNA, and medium alone treatment for the defined time. The protein of phospho-Akt was increased in the EGF group compared to the control group, and the expression of phosphor-Akt was notably changed when compared with the EGF+*eIF5A* siRNA and *eIF5A* siRNA groups. No differences were noted between the EGF+*eIF5A* siRNA and EGF+negative siRNA groups. 

### The effect of LY294002 on *eIF5A*, *MMP9*, and *PCNA* expression and HCEpiC apoptosis

**Figure 4:**
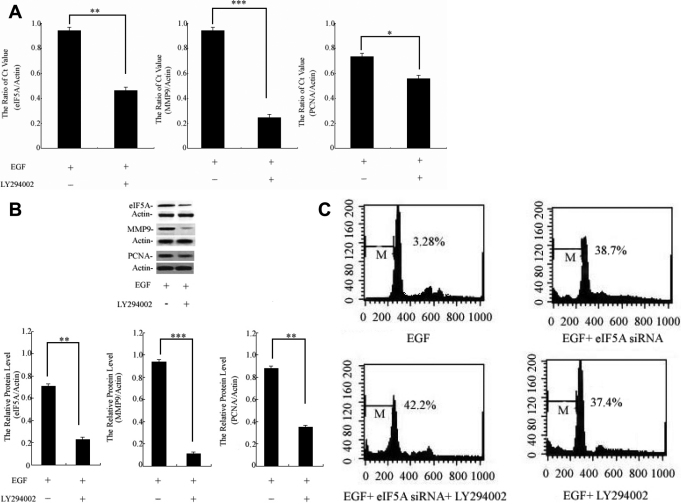
*eIF5A*, *MMP9*, and *PCNA* expression in HCEpiCs treated with EGF (control) or EGF+LY294002 were assessed in this experiment. **A**: *eIF5A*, *MMP9*, and *PCNA* gene expression was detected by real-time PCR. The graph shows the relative *eIF5A*, *MMP9*, and *PCNA* mRNA levels normalized to *ACTB*. **p<0.01 versus control group, ***p<0.001 versus control group. **B**: The expression of eIF5A, MMP9, and PCNA protein was measured by using western blot analysis. The graph shows the relative eIF5A, MMP9, and PCNA protein levels normalized to β-actin. Results are presented as the mean±standard deviation of three independent experiments (n=3), each conducted in triplicate. (**) were highly significant when compared with control group, in both cases p-value < 0.01, three asterisks indicate p-value < 0.001 with the Student t test. **C**: HCEpiCs were treated with EGF, EGF+*eIF5A* siRNA, EGF+*eIF5A* siRNA+LY294002, and EGF+LY294002. HCEpiCs were treated with 10 µM LY294002 (PI3-k inhibitor) for 18 h; at 48 h post infection, cells were stimulated with EGF. After further incubation for 18 h, cells were subjected to flow cytometric analysis.

In this experiment we examined the effect of PI3-k/Akt inhibitor LY294002 on *eIF5A*, *MMP9*, *PCNA*, and HCEpiC apoptosis by comparing HCEpiCs treated with EGF+LY294002 to a control (EGF alone). HCEpiCs were treated with 10 µM LY294002 (PI3-k inhibitor) for 18 h, and cells were then stimulated with 10 ng/ml EGF for another 24 h. Real-time PCR results showed that treatment with LY294002 caused 51%, 78%, and 29% inhibition of *eIF5A*, *MMP9*, and *PCNA* mRNA expression, respectively, when compared to the control ( Figure 4A). Western blot results showed that LY294002 treatment caused 69%, 88%, and 65% inhibition of eIF5A, MMP9, and PCNA protein expression, respectively, when compared with the control (Figure 4B). HCEpiC apoptosis, subjected to flow cytometric analysis, showed the least number of apoptosis cells with EGF treatment when compared with other groups whereas the number of apoptotic cells by treating cells with EGF + *eIF5A* siRNA, EGF + *eIF5A* siRNA + LY294002, and EGF + LY294002 considerably increased the number of subG1 cells (Figure 4C). These results suggest that EGF-induced HCEpiC proliferation may primarily be associated with upregulation of *eIF5A* expression via activation of the PI3-k/Akt signaling pathway.

## Discussion

The renewal of the corneal epithelium is a complex process that includes the migration, proliferation, and differentiation of epithelial cells, which maintain a healthy condition of the corneal epithelium through a dynamic wound-healing process [8]. This process is largely controlled by intercellular signaling pathways through activation of a growth factor. Our data indicate that serum-containing growth factors, such as EGF, stimulate corneal epithelial cell growth by upregulation of *eIF5A* expression. We investigated the relationship between the effects of EGF on the production of eIF5A. The results showed that the expression of eIF5A was significantly increased in HCEpiCs treated with EGF (Figure 1). This finding suggests that *eIF5A* may play an important role in the growth of EGF-treated HCEpiCs.

eIF5A is considered a nucleocytoplasmic shuttle protein, which is a multifunctional cellular protein expressed in a wide range of tissues and cell types, including lymphocytes, endothelial cells, dendritic cells, and platelets [9]. Several studies have also found a role for *eIF5A* involvement in cell proliferation, and more recently it has been implicated in the regulation of apoptosis [10]. Overexpression of *eIF5A* has been found to induce hepatocellular carcinoma proliferation [11] and skeletal stem cell differentiation [12]. To determine whether EGF correlates with the proliferative activity occurring in HCEpiC monolayers in vitro, we applied siRNA to knock down the expression of *eIF5A* in corneal epithelial cells. This is the first time this type of experiment has been carried out. We attempted to define the migratory mechanism of EGF-induced *eIF5A* expression via the MMP9 pathway. As shown in Figure 2A,B *MMP9* expression increases with EGF treatment but significantly decreases with *eIF5A* siRNA treatment. Another aspect of re-epithelialization is the proliferation of epithelial cells behind the migrating wound front. In our study, we showed that *PCNA* increased expression in EGF treatment, while the expression of *PCNA* treated with EGF+*eIF5A* negative cells was greater than in treatment with EGF+*eIF5A* siRNA cells (Figure 2C,D). The cell proliferation assay further revealed that DNA synthesis significantly increased in EGF treatment. A statistically significant increase was noted in the EGF+negative siRNA treatment group, whereas the number of cells were lowest in the *eIF5A* siRNA group. Our results indicate that EGF promotes corneal epithelial proliferation and induces upregulation of *eIF5A* expression, which affect *MMP9* and *PCNA* expression, and HCEpiC proliferation.

Serum-containing growth factors can induce transactivation of receptor tyrosine kinases and activate the PI3-k/Akt signaling pathway [13]. PI3-k is a heterodimeric cytoplasmic enzyme that physically associates with tyrosine-phosphorylated membrane-bound cellular proteins via the Src homology 2 (SH2) domain of itself (85 kDa regulatory subunit). Akt is one of the PI3-k effectors that play an important role in mediating transformation and anti-apoptotic effects [14,15]. These findings lead to the hypothesis that translocation and membrane localization of PI3-k are necessary for its activation in vivo. Several other studies have also reported that PI3-k/Akt has a positive role in the EGF-induced proliferation of corneal epithelial cells [16]. Our data show that EGF can activate the PI3-k/Akt signal transduction pathway, but the expression of p*-Akt protein does not appear to change between EGF+negative siRNA and EGF+*eIF5A* siRNA groups. Since eIF5A has no effect on the expression of p*-Akt (Figure 3), we conclude that the PI3-k/Akt signaling pathway is downstream for EGF but not for *eIF5A*.

Further studies are being performed to understand how the PI3-k/Akt signaling pathway is involved in EGF-induced corneal epithelial cell proliferation. Figure 4A,B shows that pretreatment with LY294002 inhibits the expression level of *eIF5A*, *MMP9*, and *PCNA*, suggesting that eIF5A, MMP9, and PCNA are possible substrates for the PI3-k/Akt signaling pathway in EGF-induced HCEpiC proliferation. Moreover, our data indicate that synergistic treatment with *eIF5A* siRNA and LY294002 significantly increases apoptotic HCEpiC death.

In summary, we have demonstrated for the first time that EGF induces HCEpiC proliferation via upregulation of *eIF5A* expression. The effect of *eIF5A* is accomplished in corneal epithelial cells through activation of the PI3-k/Akt signaling pathway, suggesting that upregulation of *eIF5A* is importance for EGF to elicit control of corneal epithelial cell growth and fate.
